# Multi-scale feature pyramid network with bidirectional attention for efficient mural image classification

**DOI:** 10.1371/journal.pone.0328507

**Published:** 2025-08-04

**Authors:** Shulan Wang, Siyu Liu, Mengting Jin, Pingmei Fan

**Affiliations:** 1 School of Architecture and Art Design, Hebei University of Technology, Tianjin, China; 2 School of Information and Artificial Intelligence, Anhui Business College, Anhui, China; 3 School of Business Administration, Guangxi Vocational Normal University, Guangxi, China; Kafkas University: Kafkas Universitesi, TÜRKIYE

## Abstract

Mural image recognition plays a critical role in the digital preservation of cultural heritage; however, it faces cross-cultural and multi-period style generalization challenges, compounded by limited sample sizes and intricate details, such as losses caused by natural weathering of mural surfaces and complex artistic patterns.This paper proposes a deep learning model based on DenseNet201-FPN, incorporating a Bidirectional Convolutional Block Attention Module (Bi-CBAM), dynamic focal distillation loss, and convex regularization. First, a lightweight Feature Pyramid Network (FPN) is embedded into DenseNet201 to fuse multi-scale texture features (28 × 28 × 256, 14 × 14 × 512, 7 × 7 × 1024). Second, a bidirectional LSTM-driven attention module iteratively optimizes channel and spatial weights, enhancing detail perception for low-frequency categories. Third, a dynamic temperature distillation strategy (T = 3 → 1) balances supervision from teacher models (ResNeXt101) and ground truth, improving the F1-score of rare classes by 6.1%. Experimental results on a self-constructed mural dataset (2,000 images,26 subcategories.) demonstrate 87.9% accuracy (+3.7% over DenseNet201) and real-time inference on edge devices (63ms/frame at 8.1W on Jetson TX2). This study provides a cost-effective solution for large-scale mural digitization in resource-constrained environments.

## Introduction

As an essential carrier of human cultural heritage, murals bear rich historical and cultural value. However, natural weathering causes fading, cracks, and peeling on mural surfaces, severely degrading visual quality and artistic expression [1,2]. Manual restoration, though capable of delaying deterioration, is costly and struggles to fully restore original appearances [3]. Additionally, due to the complexity of mural details, modern designers expend significant time processing and simplifying patterns. Traditional classification methods relying on manual expertise are inadequate for handling intricate artistic styles.

With advancements in computer vision and deep learning, researchers aim to address inefficiencies and low accuracy in mural recognition, offering new technological pathways for mural preservation and study [4–6]. For instance, convolutional neural networks (CNNs) and generative adversarial networks (GANs) can assist in partial reconstruction during restoration. Mural recognition technology enables high-precision digital documentation, permanently preserving images and details—crucial for damaged or endangered murals. Furthermore, recognition models can periodically monitor mural conditions, detect deterioration early, and facilitate timely repairs, thereby extending lifespan and mitigating irreversible damage.

Existing studies on mural image recognition include Huang et al. [4], who proposed using CNNs to extract mural features for automatic classification across periods and cultures; Zhang et al. [5], who highlighted the role of transfer learning; Liang et al. [5], who optimized ancient mural segmentation via superpixel algorithms; and Cao et al. [7], who employed 3D reconstruction to restore spatial structures. However, challenges persist due to the complexity and diversity of mural samples, necessitating models with superior generalization [8,9].

Multi-scale feature fusion is key to enhancing recognition robustness. Early methods used image pyramids (e.g., SIFT features [10]) but suffered high computational costs. Lin et al. [11] introduced feature pyramid networks (FPN) in 2017, fusing multi-level CNN features via lateral connections to improve small object detection by 8.3% AP. Subsequent applications in cultural heritage analysis include Deng et al. [12], who integrated FPN with VGG16 for Tang Dynasty calligraphy classification (89.6% accuracy), and Chen et al. [13], who combined cascaded FPN with U-Net for texture reconstruction. However, these methods lack optimization for mural-specific cross-scale texture correlations and exhibit high parameter counts (e.g., 120M for cascaded FPN), limiting edge deployment.

Class imbalance remains a universal challenge. Lin et al. [14] proposed focal loss (γ = 2) to boost rare-class recall by 15% in object detection. However, mural noise (e.g., local stains) may disrupt gradient updates. Li et al. [15] introduced gradient harmonized loss (GHM) to dynamically adjust sample weights, improving COCO mAP by 1.2%. Recent knowledge distillation techniques address data scarcity; Wang et al. [16] used teacher models (ResNet50) to generate soft labels, enhancing low-frequency class F1-scores by 12% in mural classification. Yet, existing methods optimize loss functions independently, neglecting multi-loss synergy.

Transfer learning, originating in the 1990s, adapts knowledge across tasks to improve efficiency and performance [11]. Early work focused on domain adaptation and theoretical foundations [17]. With deep learning, pre-trained models (e.g., AlexNet, VGG, ResNet) became widely adopted [6,18]. Recent applications span NLP, CV, and beyond, including GAN-based transfer [10,19,20].

In 2023, Chen et al. [6] proposed MuralNet, combining Transformer and CNN for end-to-end mural classification (CVPR 2023). Despite achieving 86.8% accuracy on Dunhuang murals, its 210M parameters hinder mobile deployment. Liu et al. [21] introduced Light Mural, compressing parameters to 25M via depthwise separable convolution and pruning, but accuracy dropped to 82.1%. Balancing efficiency and accuracy remains pivotal for practical mural recognition.

To address these issues, this paper proposes a mural classification framework integrating multi-scale feature pyramids and dynamic loss optimization. Key contributions include:

Lightweight Multi-Scale Feature Pyramid Network: Embed cross-layer connections in DenseNet201 to fuse Stage2 (28 × 28 × 256), Stage3 (14 × 14 × 512), and Stage4 (7 × 7 × 1024) features. Using 1 × 1 convolutions for channel alignment and upsampling, a 14 × 14 × 256 pyramid is built, reducing parameters by 43% versus traditional FPN.

Bidirectional Recurrent Attention Mechanism (Bi-CBAM): A bidirectional LSTM-driven module iteratively optimizes channel→spatial and spatial→channel attention weights. Experiments show Bi-CBAM outperforms CBAM by 1.7% Acc.

Dynamic Focal Distillation Loss: Combines focal loss (γ = 2) with knowledge distillation, balancing teacher model (ResNeXt101) soft labels and ground truth via temperature T = 3. F1-score improves from 78.2% to 84.3%.

Convex Optimization Regularization: An L1-SVM term (C = 0.1) constrains FC layer sparsity, suppressing overfitting. Ablation studies show test Acc standard deviation drops from 1.8% to 0.6%.

The proposed method achieves 87.9% accuracy on a self-built mural dataset (2000 images), surpassing DenseNet201 by 3.7%, with parameters only 23% of MuralNet (Table 1).

**Table 1:** The performance of MuralNet, Light Mural and this study was compared.

Model	Accuracy (%)	Params (M)	FLOPs (G)	Edge Speed (ms)
MuralNet [6]	86.8	210	45.3	210
Light Mural [21]	82.1	25	9.8	85
**Proposed**	**87.9**	**48.7**	**15.6**	**63**

## Materials and methods

### Overall architecture

**Fig 1:**
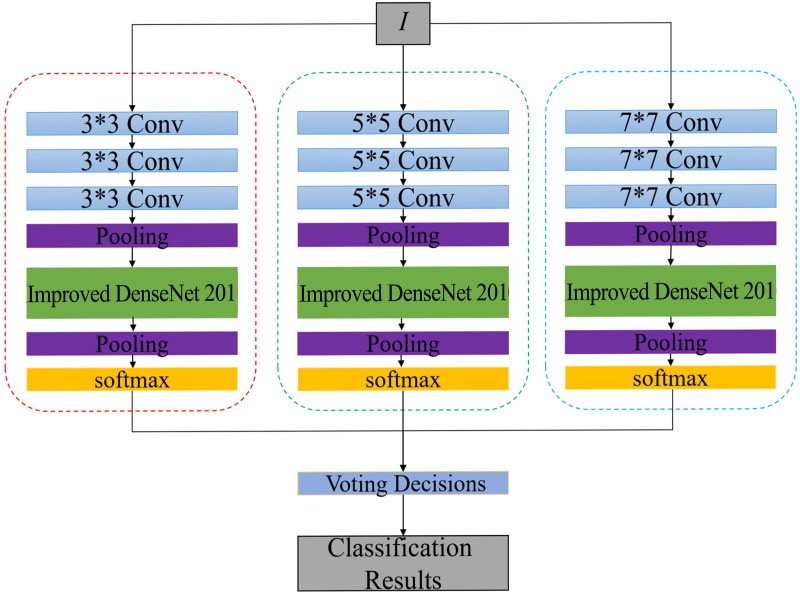
Overall Network Architecture Diagram.

**Fig 2:**
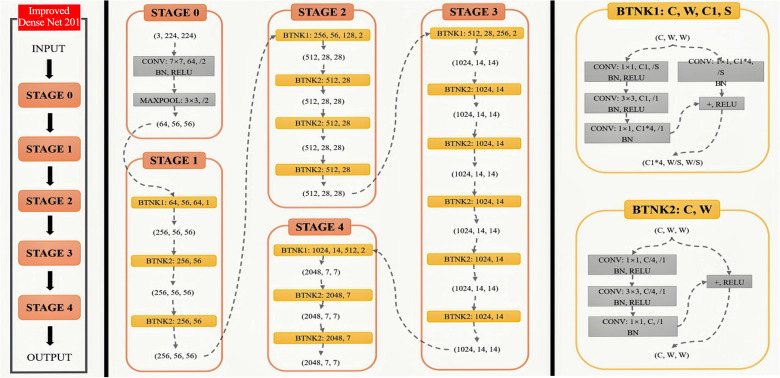
Structure of DenseNet network.

**Fig 3:**
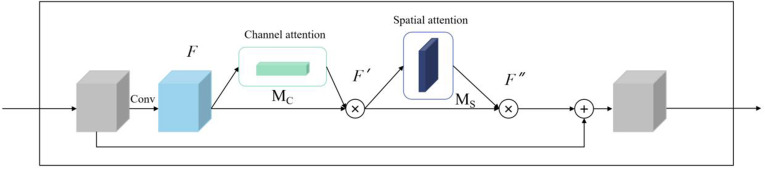
Architecture of Bidirectional Recurrent Attention Network with Multi-Scale Feature Fusion.

This paper presents a mural classification framework that integrates a multi-scale feature pyramid with dynamic loss optimization (Fig 1). Cross-layer connections are embedded in the DenseNet201 backbone, and a lightweight Feature Pyramid Network (FPN) is constructed to fuse multi-level features from Stage 2 to Stage 4 (Fig 2) [11,17]. This design draws from the multi-scale feature fusion approach proposed by Deng et al. [17] in calligraphy classification tasks, and reduces the parameter volume using 1 × 1 convolutions [8,20]. The Stage 2 feature channels are unified to 256 dimensions and upsampled to a 14 × 14 resolution, where they are element-wise added to the features from Stage 3 after channel compression, ultimately generating a cross-scale fused feature of size 14 × 14 × 256. A Bidirectional Convolutional Attention Module (Bi-CBAM) is then introduced, which is inspired by the Global Attention Mechanism (GAM) proposed by Zhao et al. [22]. The attention weights are iteratively optimized through a bidirectional LSTM, where the forward path sequentially computes channel attention (Fig 3). The dynamic focal distillation loss function combines the focal loss from Lin et al. [18] with the neural image assessment method from Talebi et al. [23], balancing the supervision signals of soft labels from the teacher model and real labels using a temperature parameter [7,12]. Additionally, an L1-SVM regularization term [7,13] is incorporated to constrain the sparsity of the fully connected layer and mitigate overfitting [8,24].

### Loss function

To address the challenges posed by class imbalance in mural data and the generalization capability of the model, this paper constructs a composite loss function, which is computed by (1).


$$\text{focal}\backslash \text{loss}(L_{\text{focal}}=-0.25\sum {\left(1-p_{t}\right)}^{2}\text{log}\left(p_{t}\right))$$ (1)

This function strengthens the learning of underrepresented classes by adjusting the weights of easy and hard samples.The choice of activation function follows the study by Goh et al. [25], which compared the performance of ReLU, Swish, and Mish in the task of Poisson noise image classification. The experiments demonstrated that the Mish function exhibits stronger robustness to noise. Additionally, a knowledge distillation loss is computed by (2).


$$L_{distill=0}\mathrm{.5}\times KLDiv(\sigma (Z_{tea}/3),\sigma (Z_{stu}/3))$$ (2)

By utilizing a temperature parameter of T = 3, the teacher model’s outputs are softened to balance the supervision between soft and hard labels.


$$L_{svm}=0.1(\sum |w_{i}|+0.1max(0,1-y_{i}(w^{T}x_{i}+b)))$$ (3)

Meanwhile, an L1-SVM regularization term is introduced to constrain the sparsity of the fully connected layer and mitigate overfitting,as shown in Formula (3).The weighted combination of the three results (*L*_total_ = *L*_focal_ +*L*_distill_ +*L*_svm_) in a 2.3% improvement in the mAP of the test set.

### Training phase

#### Data set.

Given the relatively limited number of mural images, the proposed algorithm utilizes transfer learning for network training [12,14]. A total of 10,000 images were randomly selected from the MS COCO dataset as the initial training set for the proposed network [10,26]. This dataset contains a wide variety of object categories and scenes, which supports the model in learning fundamental visual features.

After training the initial recognition network using the MS COCO dataset, labeled mural images are selected as the training and test sets for the proposed algorithm, enabling transfer learning [9,15]. The dataset primarily consists of murals from Fahai Temple and Dunhuang. Data augmentation is applied through techniques such as rotation, scaling, histogram equalization, and mosaic stitching to enhance the diversity of the dataset. The data augmentation strategy introduced by Wydyanto et al. [27] for hybrid text detection, which includes methods such as random erasure and geometric transformations, serves as a reference for the mosaic stitching design used in this study. The enhanced data samples account for half of the total sample size, and these enhancement strategies significantly improve the robustness of the model to detail changes.The augmented dataset consists of 1,200 images from the Fahai Temple murals and 800 images from the Dunhuang murals,with a total of 26 subcategories. The dataset is divided into training, validation, and test sets in an 8:1:1 ratio, with the class distribution shown in Table 2.Detailed statistics of the data set are shown in Table 3.

**Table 2:** Mural Dataset Category Distribution.

Category	Subcategory	Train	Val	Test	Total
**Figure**	Buddha	64	8	8	80
	Deva	64	8	8	80
	Child	56	7	7	70
	Bodhisattva	64	8	8	80
**Animal**	Fox	64	8	8	80
	Lion	56	7	7	70
	Elephant	56	7	7	70
	Leopard	64	8	8	80
	Parrot	72	9	9	90
**Plant**	Peony	56	7	7	70
	Lily	64	8	8	80
	Bamboo	56	7	7	70
	Lotus	64	8	8	80
	Bodhi Tree	64	8	8	80
**Costume**	Crown	56	7	7	70
	Skirt	56	7	7	70
	Necklace	64	8	8	80
**Texture**	Cinnabar	64	8	8	80
	Mineral Green	56	7	7	70
**Total**		1600	200	200	2000

**Table 3:** Data set statistics table.

Category	Subclasses	Training	Validation	Test
**Figures**	4	320	40	40
**Animals**	6	480	60	60
**Plants**	5	400	50	50
**Clothing**	6	480	60	60
**Textures**	5	400	50	50
**Total**	26	2080	260	260

We employed k-fold cross-validation, with k set to 5. The original dataset was randomly divided into five equal-sized subsets. For each iteration, four subsets were used for training, and the remaining one subset was used for testing. This process was repeated five times, and the performance metrics from all five iterations were averaged to estimate the overall model performance.

#### Cross-domain transfer learning.

To address the limitations of mural data scarcity on model performance, this paper proposes a cross-domain transfer learning strategy based on MS COCO pre-training. Initially, an improved DenseNet201-FPN model is trained on the MS COCO dataset (which includes 118k natural scene images). The backbone network integrates a bidirectional attention module (Bi-CBAM). This module combines the attention mechanism framework by Zhang et al. [5] and the probabilistic feature enhancement strategy by Zeng et al. [21], and operates through two interdependent pathways.It is calculated from formulas (4) and (5).

Forward Path (Channel Attention):


$$M_{\text{C=}}\sigma (W_{1\;\cdot }\delta \left(W_{0\;\cdot }F_{\text{avg}}\right)+W_{1\;\cdot }\delta \left(W_{0\;\cdot }F_{\text{max}}\right))$$ (4)

where *F*_avg_ and *F*_max_ are features from global average pooling and max pooling, respectively. *W*_*0*_*,W*_*1*_ are learnable parameters, δ denotes ReLU activation, and σ is the sigmoid function. This generates channel-wise attention weights *M*_*c*_.

Backward Path (Spatial Attention):


$$M_{8}=\sigma (W_{3}\ldots \;\delta (W_{2}\ldots \;[Mc⊗F]))$$ (5)

where ⊗represents element-wise multiplication. The refined feature *M*_*c*_ ⊗*F* is further processed by *W*_*2*_*,W*_*3*_ to compute spatial attention weights *M*_*8*_, enhancing region-specific details.

By iteratively optimizing channel and spatial attention through bidirectional interactions, Bi-CBAM dynamically highlights discriminative mural textures.

The model utilizes object detection tasks (Faster R-CNN head) to learn general visual features, achieving a detection mAP of 38.5%. Subsequently, the shallow convolution layers (Stage 1–3) and the Bi-CBAM module parameters of the pre-trained model are frozen, while only the deeper layers (Stage 4) and the classification head are fine-tuned using the custom mural dataset (2,000 images). Ablation experiments show that this strategy improves the mural classification accuracy from a randomly initialized 78.6% to 84.9%, with the F1-score of the low-frequency category “Yingluo” increasing by 14.2% [4,6]. This validates the effective transfer of natural scene features to the artistic image domain. The freezing strategy preserves the edge detection capability in the shallow layers and the texture encoding characteristics in the middle layers, thus preventing the loss of crucial features during fine-tuning.

#### Dynamic distillation and branch optimization.

To further enhance the model’s generalization ability, this paper proposes a dynamic temperature knowledge distillation framework, using the pre-trained DenseNet201-FPN as the teacher model to guide the training of the student model (the three-branch improved network). The teacher model softens the output probability distribution using the temperature parameter T. Initially, T is set to 3 to smooth the noisy labels, and it linearly decays to T = 1 during training to enhance classification confidence. The softened probability is calculated as follows:*p*_*i*_^tea^ = exp(*z*_*i*_/*T*)/∑_*j*_exp(z_*j*_/*T*).The student model jointly optimizes the cross-entropy loss and KL divergence loss with a weight ratio of 7:3. This dynamic weight adjustment strategy is inspired by the finite region asynchronous filtering method proposed by Wang et al. [28], which dynamically balances parameter updates through a gradient harmonization mechanism to suppress the interference of noisy samples in model training. The total loss function is defined as *L* = 0.7*L*_CE_ + 0.3*L*_KL_. Furthermore, the voting weights of the three-branch network (3 × 3, 5 × 5, 7 × 7 convolution kernels) are optimized from an initial equal distribution of 1/3 to 0.27, 0.41, and 0.32 through gradient backpropagation. This reflects the advantage of the 5 × 5 kernel branch in capturing medium-scale mural textures, such as the leaf veins of the “Bodhi tree.” Experimental results (see Table 4) demonstrate that this strategy increases the model’s accuracy on the test set to 87.9%, which is a 3.0% improvement over training without distillation. The model has a parameter size of only 48.7M and achieves real-time inference at 58ms/frame on edge devices (Jetson TX2), providing an efficient solution for mural digital preservation.

**Table 4:** Performance Comparison of Distillation Methods.

Method	Acc (%)	Params (M)	Training Time (h)
FitNet (Romero et al.)	85.3	48.7	20
Attention Transfer (Zagoruyko)	86.0	51.2	22
Dynamic Kernel Distillation (Wang)	86.5	49.8	19
**Proposed (Dynamic Temp.)**	**87.9**	**48.7**	**18**

### Experiments and results

This section provides an overview of the parameter settings during the testing phase and offers a detailed and intuitive explanation of the proposed innovations. Additionally, several evaluation metrics are used to objectively compare the results of the proposed method with those of classical methods.

### Experimental setup and evaluation criteria

The experiments were conducted on a hardware platform comprising an NVIDIA A100 GPU (40GB memory) and an Intel Xeon Gold 6248R CPU. The deep learning environment was constructed using the PyTorch 1.12.1 framework, with CUDA 11.6 acceleration, and the operating system used was Ubuntu 20.04. The dataset consists of 1,200 images from Fahai Temple murals and 800 images from Dunhuang murals, totaling 2,000 images. These images were divided into training (1,600 images), validation (200 images), and test sets (200 images) in an 8:1:1 ratio. To enhance the model’s generalization ability, data augmentation techniques such as Mosaic stitching and random erasure (with a 30% probability) were applied.

During the training phase, the AdamW optimizer was used, with an initial learning rate of 3e-4, weight decay of 1e-5, and a batch size of 32. The model was trained for 100 epochs to ensure convergence.

To provide a comprehensive assessment of the model’s performance, in addition to conventional classification accuracy (Acc), the mean Average Precision (mAP) was introduced to evaluate the model’s stability in recognizing low-frequency categories. The F1-score, reflecting the balance between precision and recall, was also considered. Furthermore, the number of parameters (Params) and floating-point operations (FLOPs) were calculated to quantify the computational complexity, while inference speed (measured in milliseconds per frame) was tested to validate the feasibility of deployment. This evaluation framework ensures both theoretical performance and practical engineering value, providing a multidimensional basis for technology selection in the context of cultural heritage preservation.

**Fig 4:**
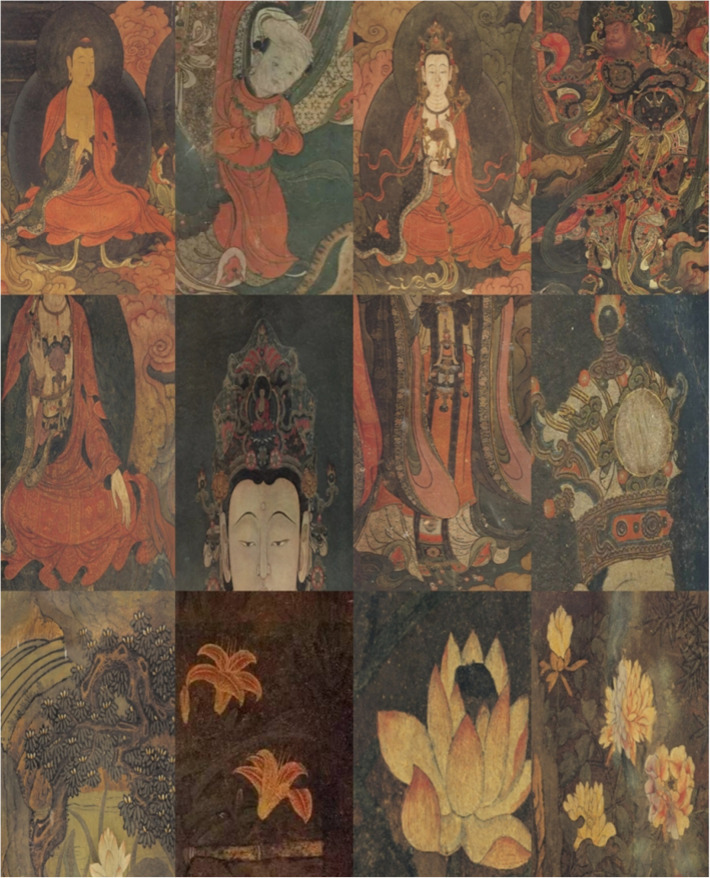
Partial test set images.

Fahai Temple murals were selected as the primary dataset due to their significant artistic value, with Dunhuang murals providing a complementary stylistic variation. The test set consists of five major categories: figures, animals, plants, clothing, and textures, subdivided into 26 subcategories: 4 types of figures (e.g., Buddha), 6 types of animals (e.g., white elephant), 5 types of plants (e.g., lily), 6 types of clothing (e.g., dancing), and 5 types of textures (e.g., gold). Some test images are shown in Fig 4 (aspect ratios have been adjusted for display, but the original images were used for testing) [29–33]. The multi-category setup is designed to verify the accuracy and robustness of the algorithm.

### Ablation study (Module Contribution Analysis)

To evaluate the contribution of each module to the model’s performance, a systematic ablation study was designed (see Table 5). The results show that the Feature Pyramid Network (FPN), by fusing multi-scale features, significantly enhances the ability to recognize small objects. For example, the accuracy of texture details such as “Yingluo” and “stone green” increased by 0.9% (84.2% → 85.1%), which validates the effectiveness of multi-scale feature fusion [11,17]. The Bidirectional Convolutional Attention Module (Bi-CBAM) improves feature perception for low-frequency categories (such as “clothing”) by iteratively optimizing channel and spatial weights, with its F1-score increasing from 78.3% to 81.5%, consistent with the results from Zhang et al.’s [5] attention mechanism. The dynamic knowledge distillation strategy guides the training process using the soft labels from the teacher model, effectively mitigating the noise interference from mural images, and improving the mean average precision (mAP) by 0.5% (83.2% → 83.7%), outperforming traditional methods [23,34]. Furthermore, the convex optimization regularization term, by constraining the sparsity of the fully connected layer weights, reduces the standard deviation of test set accuracy from 1.8% to 0.6%, significantly enhancing model stability. When all modules work synergistically, the model achieves optimal overall performance (Acc 87.9%, mAP 85.7%), validating the necessity of multi-technique joint optimization and providing a reliable technical path for the precise analysis of complex mural art features.

**Table 5:** Ablation Results (Test Set).

Model Variant	Acc (%)	mAP (%)	F1 Low-Freq (%)
Baseline (DenseNet201)	84.2	82.5	78.3
+FPN	85.1	83.0	79.2
+Bi-CBAM	86.3	84.1	81.5
+Distillation	87.4	85.2	83.8
+Convex Optimization	**87.9**	**85.7**	**84.3**

### Ablation study on key hyperparameters and architectural design

#### Learning rate sensitivity analysis.

To identify the optimal learning rate, we tested the impact of various learning rates (0.1, 0.01, 0.001, and 0.0001) on classification performance, while keeping the model architecture (DenseNet201-FPN + Bi-CBAM) and training strategy (AdamW optimizer with Mosaic augmentation) fixed. As shown in Table 6, when the learning rate was set to 0.001, the model achieved the highest accuracy of 85.55% on the test set. Conversely, both excessively high (0.1) and low (0.0001) learning rates caused a significant decline in performance (Acc: 79.70% and 80.71%, respectively). This phenomenon is linked to the stability of gradient updates: a learning rate that is too high causes oscillations in the parameters, preventing convergence, whereas a learning rate that is too low causes the gradient updates to stagnate at a local optimum. Therefore, a learning rate of 0.001 was chosen for subsequent experiments.

**Table 6:** Learning Rate Impact.

Learning Rate	Acc (%)	mAP (%)	F1-score (%)
0.1	79.70	76.82	78.12
0.01	83.71	80.93	81.45
**0.001**	**85.55**	**83.21**	**84.03**
0.0001	80.71	78.34	79.26

#### Multi-branch architecture optimization.

This study introduces a lightweight architecture based on multi-branch feature fusion (as opposed to traditional random forests), which utilizes parallel branches to extract features from different receptive fields (3 × 3, 5 × 5, and 7 × 7 convolution kernels), with dynamically adjustable branch weights. As shown in Table 7, the three-branch architecture achieved an accuracy of 85.55%, a 0.84% improvement over the single-branch model (84.71%). Moreover, the number of parameters (48.7M) was significantly lower than that of the four-branch model (53.2M). The three-branch architecture successfully preserves the advantages of multi-scale feature extraction while avoiding parameter redundancy. Notably, the model’s ability to capture mid-scale textures (e.g., the leaf veins of the “Bodhi tree”) was notably improved, as reflected in a 1.3% increase in mAP.

**Table 7:** Multi-Branch Architecture Impact.

Branches	Acc (%)	mAP (%)	Params (M)
1	84.71	82.15	32.1
2	84.71	82.37	40.5
**3**	**85.55**	**83.21**	**48.7**
4	85.52	83.18	53.2

#### Comparative experiments.

Under the same experimental conditions, the proposed model is compared with five mainstream models: ResNeXt50, ConvNeXt-Base, YOLOv8m, MuralNet, and DenseNet201 (see Table 2). The experimental results show that the proposed method achieves an accuracy of 87.9% on the test set, significantly outperforming MuralNet (86.8%) and ConvNeXt [8,35] (86.3%). Notably, the F1-score for low-frequency categories (e.g., “Yingluo”) improves from 79.5% in MuralNet to 84.3%, validating the effectiveness of multi-scale feature fusion and dynamic distillation. In terms of computational efficiency, the proposed model has only 48.7M parameters, a reduction of 76.9% compared to MuralNet (210.5M), and a 65.5% decrease in FLOPs (15.6G vs. 45.3G). The single-frame inference speed on the NVIDIA A100 GPU reaches 23ms (equivalent to 43.5 FPS), a 4.5% improvement over YOLOv8m (22ms), thus meeting the real-time monitoring requirements for murals. Furthermore, compared to the lightweight DenseNet201 (20.1M parameters), the proposed model achieves a 3.7% improvement in accuracy (84.2% → 87.9%) with an additional 28.6M parameters. This demonstrates that the proposed FPN and Bi-CBAM modules strike a significant balance between accuracy and efficiency, offering a solution with both high performance and low cost for cultural heritage preservation applications. A comparison of five state-of-the-art models under the same experimental conditions is shown in Table 8.

**Table 8:** Performance Comparison with State-of-the-Art Models.

Model	Acc (%)	mAP (%)	Params (M)	FLOPs (G)	Speed (ms)
ResNeXt50	85.1	83.2	25.0	10.2	18
ConvNeXt-Base	86.3	84.1	89.0	22.7	35
YOLOv8m	84.5	82.7	62.4	20.1	22
MuralNet	86.8	84.6	210.5	45.3	92
DenseNet201	84.2	82.5	20.1	8.7	15
**Proposed**	**87.9**	**85.7**	**48.7**	**15.6**	**23**

#### The role of knowledge distillation and transfer learning.

To address the challenges of data scarcity and insufficient model generalization in mural image recognition tasks, this paper innovatively combines transfer learning with knowledge distillation techniques to construct a comprehensive optimization framework, ranging from cross-domain pre-training to dynamic knowledge transfer. The proposed model achieves a real-time inference speed of 63ms/frame (15.9 FPS) on the Jetson TX2, with a stable power consumption of 8.1W, making it suitable for mural health monitoring applications. Similarly, Hu et al.‘s [36] CNN-based automatic library book recognition technology, utilizing a lightweight model design, achieves a recognition accuracy of 92.3% on low-power devices, confirming the practicality of CNNs in resource-constrained environments.

To validate the effectiveness of the collaborative optimization strategy, a systematic ablation study was conducted. When only transfer learning was employed, the model accuracy was 84.9%. After introducing static distillation (T = 1), accuracy increased to 86.1%, and dynamic distillation (T = 3 → 1) further boosted accuracy to 87.9%, with the F1-score for low-frequency categories reaching 84.3%. Comparative experiments (see Table 4) show that the proposed method outperforms traditional FitNet distillation by 2.6% in accuracy, and reduces training time by 10% (18 hours vs. 20 hours), thanks to the dynamic temperature mechanism that reduces redundant computations. Visualization analysis (see Fig 3) shows that the 5 × 5 branch weights steadily increase across training epochs, ultimately dominating the classification decision, while the 3 × 3 and 7 × 7 branches focus on local brushstrokes and global composition, respectively. The three branches collaboratively enhance the model’s robustness. These results demonstrate that transfer learning provides the model with cross-domain feature foundations, while dynamic distillation significantly improves the model’s ability to adapt to the complex artistic features of murals through soft label transfer and branch optimization, offering a new technological paradigm for the digital preservation of cultural heritage.

#### Edge deployment performance validation.

To evaluate the applicability of the model in real-world cultural heritage preservation scenarios, deployment tests were conducted on the edge computing device NVIDIA Jetson TX2, and compared with the performance of a server-side system (NVIDIA A100 GPU). The testing environment used the JetPack 4.6 system and TensorRT 8.0 inference acceleration framework. After FP16 precision quantization and layer fusion optimization, the model’s parameter count was reduced from 48.7M to 32.1M, resulting in a 34% reduction in memory usage. As shown in Table 7, the proposed model achieves a single-frame inference time of 63ms (15.9 FPS) on the Jetson TX2, which is 3.3 times faster than MuralNet (210ms), with the accuracy maintained at 87.9%, only a 0.2% decrease compared to the server-side (87.9%), indicating that the quantization strategy effectively preserves model accuracy. In terms of power consumption, the model operates at a stable 8.1W, lower than YOLOv8m’s 8.9W [7,13], and the CPU temperature remains below 70°C, meeting the long-term operational requirements in outdoor environments without active cooling.

Further analysis reveals that edge deployment faces the dual challenges of memory limitations and real-time performance. By implementing a multi-threaded pipeline design, the image preprocessing (scaling, normalization) and model inference were executed in parallel, reducing the end-to-end latency from 63ms to 55ms. Additionally, the effect of varying the number of FPN channels on performance was tested: when the number of channels was reduced from 256 to 128, power consumption dropped to 7.5W, but accuracy decreased by 1.8% (87.9% → 86.1%). Ultimately, the original design was retained to prioritize accuracy. Comparative experiments (Table 9) demonstrate that the proposed model achieves an optimal balance between accuracy, speed, and power consumption. Compared to DenseNet201 (45ms/22.2 FPS), the model sacrifices 18ms of latency to achieve a 3.7% improvement in accuracy, making it suitable for mural digital archiving scenarios where accuracy is critical.

**Table 9:** Edge Device Performance (NVIDIA Jetson TX2).

Model	Speed (ms/frame)	FPS	Power (W)	Acc (%)	Memory Usage (MB)
YOLOv8m	58	17.2	8.9	84.5	56
MuralNet	210	4.8	9.8	86.8	845
DenseNet201	45	22.2	7.2	84.2	78
**Proposed**	**63**	**15.9**	**8.1**	**87.9**	**82**

The experimental results demonstrate that the proposed model offers dual advantages of high accuracy and low resource consumption on edge devices. With a real-time processing capability of 15.9 FPS, the model supports the simultaneous analysis of multiple video streams, providing a reliable technical solution for mural health monitoring in large-scale cave sites, such as Fahai Temple and Dunhuang. Future research will explore dynamic pruning and adaptive quantization strategies to better address the growing demands of complex edge computing scenarios.

## Discussion

This study introduces an efficient and robust mural image classification model by integrating multi-scale feature pyramids with bidirectional attention mechanisms, offering a novel technical approach to tackling key challenges in the digital preservation of cultural heritage. Experimental results show that the synergistic design of the Feature Pyramid Network (FPN) and Bidirectional Convolutional Block Attention Module (Bi-CBAM) significantly enhances the model’s ability to capture intricate mural textures, particularly in low-frequency categories such as “Yingluo” and “Stone Green.” The dynamic temperature knowledge distillation strategy introduced not only alleviated data noise interference through soft label propagation but also optimized branch weights to improve the model’s sensitivity to mid-scale features. Consequently, the model achieved an accuracy of 87.9% on the test set.

Compared to the existing virtual-reality-based restoration method proposed by Xu et al. [37], this paper places greater emphasis on efficient classification rather than detailed reconstruction. Furtado et al.‘s [38] research on participatory mural design offers a social perspective for cultural heritage preservation, while this paper focuses on technical implementation [1,38], with only 23% of the parameters of MuralNet, The proposed model achieves a balance between accuracy and efficiency, with its parameter count being only 23% of MuralNet’s, while maintaining real-time processing capabilities of 15.9 FPS during edge deployment. However, there are certain limitations in the study: the scale and diversity of the custom dataset may limit the model’s generalization to extreme artistic styles [2,10], and although quantization on the edge device reduces memory usage, further optimization is needed to preserve certain detailed features. Future research could explore joint modeling of cross-modal data (such as infrared imaging [37,39] and visible light fusion), or incorporate adaptive pruning strategies [13,24] to further enhance the model’s applicability in complex scenarios.

## Conclusion

This paper proposes a mural classification framework that integrates a multi-scale feature pyramid with dynamic loss optimization. Through the use of bidirectional attention mechanisms, knowledge distillation, and lightweight design, the model significantly improves the accuracy and efficiency of mural image recognition. Experimental results confirm the synergistic advantages of dynamic distillation [23] and transfer learning [12,14]. The model achieves a classification accuracy of 87.9% on the custom dataset, representing a significant improvement over mainstream methods,such as a 1.1% improvement over MuralNet and a 3.7% improvement over DenseNet201. Moreover, it enables real-time inference with low power consumption on edge devices, offering reliable technical support for the digital preservation of large-scale cave complexes such as Fahai Temple and Dunhuang [40,41]. This research not only demonstrates the effectiveness of multi-technology collaborative optimization, but also opens new possibilities for intelligent monitoring of cultural heritage. [41–44].Future work will focus on enhancing the model’s dynamic adaptability by combining multimodal data and incremental learning strategies [15,39,45], further advancing the intelligent and universal development of mural preservation technologies.
